# Evaluation of the Degradation Properties of Plasma Electrolytically Oxidized Mg Alloy AZ31 Using Fluid Dynamic Accelerated Tests for Biodegradable Implants

**DOI:** 10.3390/jfb15120366

**Published:** 2024-12-03

**Authors:** Muhammad Saqib, Kerstin Kremmer, Joerg Opitz, Michael Schneider, Natalia Beshchasna

**Affiliations:** 1 Department of Bio and Nanotechnology, Fraunhofer Institute for Ceramic Technologies and Systems IKTS, Maria-Reiche-Strasse 2, 01109 Dresden, Germany; joerg.opitz@ikts.fraunhofer.de; 2 Department of Electrochemistry, Fraunhofer Institute for Ceramic Technologies and Systems IKTS, Winterbergstrasse 28, 01277 Dresden, Germany; kerstin.kremmer@ikts.fraunhofer.de (K.K.); michael.schneider@ikts.fraunhofer.de (M.S.)

**Keywords:** plasma electrolytic oxidation, biodegradable implant, corrosion, accelerated degradation test, fluid dynamic test, resomer, Mg alloy

## Abstract

Magnesium alloys are promising biodegradable implant materials due to their excellent biocompatibility and non-toxicity. However, their poor corrosion resistance limits their application in vivo. Plasma electrolytic oxidation (PEO) is a powerful technique to improve the corrosion resistance of magnesium alloys. In this study, we present the accelerated degradation of PEO-treated AZ31 samples using a fluid dynamic test. The samples were prepared using different concentrations of KOH as an electrolyte along with NaSiO_3_. The anodizing time and the biasing time were optimized to obtain the increased corrosion resistance. The analysis of the degraded samples using microscopy, SEM EDX measurements, and by calculating mass loss and corrosion rates showed a significant increase in the corrosion resistance after the polymer (Resomer© LG 855 S) coating was applied to the anodized samples. The results confirm (or convince) that PEO treatment is an effective way to improve the corrosion resistance of AZ31 magnesium alloy. The fluid dynamic test can be used as an accelerated degradation test for biodegradable alloys in simulated body fluids at a physiological temperature. The polymer coating further improves the corrosion resistance of the PEO-treated AZ31 samples.

## 1. Introduction

We are living in the age of the scientific revolution, where most body parts can be replaced or supported by implants. According to the patient’s medical condition, a permanent or a temporary implant can be used. In recent decades, biodegradable implants have attracted more attention from researchers due to the issues associated with permanent metallic implants (e.g., thrombosis, physical irritation, restenosis, etc.) and also their inability to adapt to the growth of and changes in the human body [1,2,3].

Recently, as biodegradable materials for implantation, magnesium and its alloys have gained popularity because of their improved biocompatibility and virtually no toxicity [4,5,6,7,8,9,10,11]. Notably, among all the magnesium alloys, researchers were particularly interested in AZ31, AZ91D, Mg–Li–Ca, and WE43, and efforts have been made to employ these as bio-absorbable stents [12] and orthopedic implants [13].

Because of its lower aluminum content, improved microstructure, low fatigue, and corrosion resistance comparable to other magnesium alloys, AZ31 has received more attention [7,11,14,15,16,17]. In vivo experiments reveal that magnesium alloys have low corrosion resistance, despite their promising material features [12]. Due to their inability to adequately support the blood vessel wall, magnesium alloys are therefore not suitable for use as biodegradable stent materials. In order to improve the corrosion properties of existing magnesium alloys, it is crucial to alter their surface properties.

Various surface modification techniques have been applied to control the corrosion of Mg alloys, e.g., chemical conversion coating, physical vapor deposition, laser surface treatment, and anodic oxidation, to name a few [1].

A very powerful technique to improve the corrosion behavior of magnesium and its alloys is plasma electrolytic oxidation [18]. The process is also known under various labels, e.g., micro arc oxidation (MAO) or anodic oxidation by spark discharge (ANOF) [19]. The process is related to conventional anodizing, but the operating voltage is significantly above the breakdown potential of the passive anodic oxide layer [20]. The high operating voltage briefly ionizes the oxygen gas that is simultaneously formed at the anode. The recombination of the same appears as micro-sparks that are randomly distributed over the anode surface. The energy released in this process leads to the growth of the anodic layer, which partly consists of the crystalline high-temperature modification of magnesium oxide. Štrbák et al. reported the clearly improved corrosion stability of plasma electrolytic anodized AZ91 in chloride solution [21].

The targeted variation in the process parameters, e.g., time, voltage, and electrolytes, allows for tuning of the resulting oxide layer and its properties.

To evaluate the degradation properties of the biodegradable materials, several in vitro tests are performed (e.g., static immersion test, fluid dynamic test, and the electrochemical corrosion test) before any in vivo tests. Each in vitro test has its pros and cons and provides the different corrosion rates of the same sample. This is because of the presence and absence of influencing factors (e.g., fluid flow, electric potential, etc.) in each method. For instance, Xue et al. 2012 [22] reported that the corrosion rate of AZ31 is about five times higher for the in vitro static immersion environment than the in vivo environment. Furthermore, the corrosion rate was two times higher in PBS, whereas it was 5 times higher when they used SBF.

Koo et al., 2017 [23] compared the corrosion rates of biodegradable magnesium pins (as-drawn pure Mg, as-cast Mg-Zn-Mn, and extruded Mg-Zn-Mn) in static and dynamic conditions. They reported a corrosion rate of 1.915 ± 0.062 mm/year in the fluid dynamic conditions at 1.5 mL/min (1.6 mm/s), whereas the CR was 0.236 ± 0.022 mm/year in the static test for pure Mg. This shows the 8.1 times greater corrosion rate in the given dynamic conditions. Similarly, for as-cast Mg-Zn-Mn, the corrosion rate was 2.251 ± 0.288 mm/year and 0.527  ±  0.118mm/year in dynamic and static conditions, respectively, and for extruded Mg-Zn-Mn, the corrosion rates of 2.113 ± 0.139 mm/year and 0.188  ±  0.068 mm/year in dynamic and static conditions, respectively. The aforementioned results by Koo et al. 2017 showed the 4.3 times and 11.2 times increment in the corrosion rates of As-cast and extruded Mg-Zn-Mn, respectively, in dynamic conditions as compared to the static immersion tests. Therefore, using fluid dynamic conditions, the corrosion rates of Mg alloys can be accelerated further. Thus, this is a suitable choice for accelerated degradation tests of biodegradable alloys in the simulated body fluids at physiological temperature.

In this work, we present the accelerated degradation of the PEO-treated AZ31 samples using the fluid dynamic test. The samples were prepared using two different concentrations of KOH as an electrolyte along with NaSiO_3_. In order to achieve greater corrosion resistance, the anodizing and biasing times were also adjusted. The anodized surfaces were impregnated with Resomer©, a polymer known for its controlled degradation and non-toxic properties, offering advantages over other polymers such as PLA and PCL. The analysis of the degraded samples using microscopy and SEM-EDX measurements and by calculating mass loss and corrosion rates showed a significant increase in corrosion resistance after the polymer (resomer) coating was applied to the anodized samples.

## 2. Materials and Methods

### 2.1. Sample Preparation

The magnesium alloy AZ31 was used in this investigation. Its nominal chemical composition is shown in Table 1. The sheets were degreased and pickled with Gardoclean^®^ 5165 and Gardoclean^®^ 5491 from Chemetall (Frankfurt/M., Germany). Between the degreasing and the pickling, the sheets were cleaned with deionized water. For the handling of the sheets during the PEO process, we used a specially designed sample holder.

### 2.2. Plasma Electrolytic Oxidation Process

The PEO process was carried out in a tempered glass beaker. The anodizing process was carried out in aqueous potassium hydroxide electrolytes of varying concentrations and a fixed content of sodium silicate (see Table 2). The electrolyte conductivity was measured with 33 mS cm^−1^ (electrolyte A) and 50 mS cm^−1^ (electrolyte B). The pH value of the electrolyte was 14. The temperature was adjusted with a cryostat and was 20 °C for all experiments. The bath temperature was monitored with a thermocouple during the process. The bath was stirred during the process. An EA PS8720-15 (Elektroautomatik, Viersen, Germany) was used as a power supply. The PEO was carried out as a current-controlled pulse regime with a linear current ramp from 0 to 20 mA/cm^2^ for the first 10 s. Afterwards, the pulse regime worked with a pulse-on amplitude of 20 mA/cm^2^ and a pulse-on time of 0.5 s. The pulse-off current density was 0 mA/cm^2^ with a pulse-off time of 1 s. The total process time was 1000 s.

### 2.3. Impregnation Process

For the impregnation, a dispersion was used consisting of dichloromethane and the commercial Resomer© LG 855 S (Evonik Nutrition and Care GmbH, Essen, Germany). A total of 3% of the Resomer© was dissolved in the dichloromethane. The dip-coating parameters were as follows: withdrawing rate 4 mm/s, dipping time 1 s, room temperature. Following this, the samples were dried in a furnace at 35 °C/72 h and 80 °C/2 h. For better readability and clarity, the samples were designated as follows: Sample R (reference sample, i.e., untreated AZ31 surface), Sample 1 (anodized with electrolyte A), Sample 2 (anodized with electrolyte A and impregnated with Resomer), Sample 3 (anodized with electrolyte B), and Sample 4 (anodized with electrolyte B and impregnated with Resomer).

### 2.4. Contact Angle and Surface Wettability

The term “wettability” describes a liquid’s capacity to spread out or adhere to a solid surface. Surface wettability can have an impact on four key biological processes: (1) the adhesion of proteins and other macromolecules to the surface (conditioning); (2) the interactions between the cells of hard and soft tissues and the pre-conditioned surfaces; (3) bacterial adhesion and subsequent biofilm formation; and (4) the rate of osseointegration in the body (in vivo) [25]. The wettability is directly proportional to the contact angle. The Dataphysics OCA20 system, which makes use of industry-standard optical contact angle measuring and contour analysis techniques, was used to evaluate CAs. By using a syringe with a 0.52 mm diameter to dispense 1 L of distilled water, static water CAs were calculated. The stated CAs are averages based on a minimum of five readings per sample.

### 2.5. Optical Microscopy

A Zeiss Axioscope with brightfield and darkfield capabilities was used to capture microscopic images. A steady, vibration-free surface was used for the microscope, which was set up in a controlled lab setting. To make sure that the specimens were properly lit, the halogen light source of the microscope was adjusted to the required intensity and direction. To obtain consistent illumination, the Köhler illumination was adjusted. The proper objective lenses with 2.5× and 20× magnifications were applied. Images were taken using the Zeiss imaging program AxioVision Rel. 4.8.

### 2.6. SEM-EDX Measurements

The surface morphologies of the pure PEO layer and the impregnated PEO layer were investigated by scanning electron microscopy (Carl Zeiss Microscopy GmbH, Oberkochen, Germany).

The Environmental Scanning Electron Microscope XL30 ESEM FEG (Philips, Amsterdam, The Netherlands) was used to capture all SEM pictures and EDX measurements for fluid dynamic-accelerated degradation testing, unless otherwise noted. For SEM measurements, the accelerating voltage was adjusted to 3.0 kV with a spot size of 3 nm and 5.0 kV with a spot size of 5 nm. SEM pictures were taken before and after the degradation points of 1, 2, 4, 7, 14, 21, and 30 days for all samples. EDX measurements were also taken to determine the elemental makeup of the samples before and after degradation. For 3–5 min, the corroded samples were cleaned in ultrapure water in an ultrasonic water bath.

### 2.7. Electrochemical Measurements

The electrochemical measurements were carried out in two different solutions. The first was Hanks’ Balanced Salt Solution (HBSS) with a pH of 7.1 and the second was 0.1 M NaOH pH 12.9. For the electrochemical measurements, a common three-electrode flat cell was used at room temperature. The electrochemical tests consisted of open circuit potential measurements (ocp) over different times (0 h, 1 h and 10 h) and, afterwards, potentiostatic measurements at a potential of −1.7 V vs. SCE for 24 h. The pH value was measured at the end of each experiment. The electrochemical measurements were carried out on two different areas of the layer surfaces.

### 2.8. Fluid Dynamic Experimental Setup for In Vitro Degradation Studies

**Figure 1:**
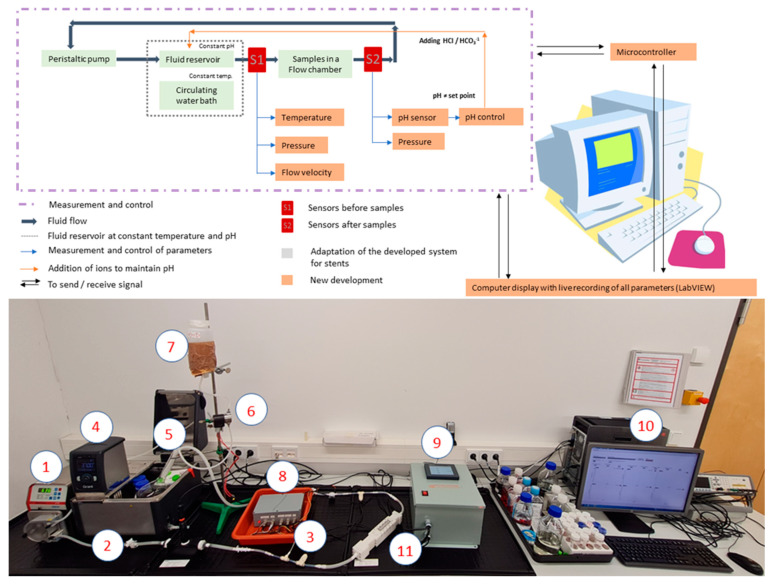
Schematic diagram of the fluid dynamic circulation setup.

The fluid dynamic experimental setup presented in [26,27] was modified for this research. Figure 1 depicts the modified fluid dynamic experimental test setup to simulate a physiological environment with pH control and parameter monitoring. The experimental setup consisted of: (1) a peristaltic pump (to mimic the heart’s pumping action); (2) puriflex tubes (as an artificial blood vessel); (3) sensors (for pressure, temperature, flow velocity, and pH); (4) a circulating water bath (to maintain a constant temperature); (5) a fluid reservoir; (6) a solenoid valve; (7) a dilute acid container; (8) a microcontroller unit box; (9) a pH controlling unit; and (10) a computer (LabVIEW program or VI to control and monitor the parameters and display the results); and (11) a flow chamber containing the samples.

With the pH being controlled, our experimental setup can offer the physiological conditions for the parameters indicated. Since the pH is crucial in the Mg’s and its alloys’ degradation and it increases with the degradation time, there is a need to regulate the pH during the experiment.

In the presented testing setup, when the pH value changes, the pH control unit of the setup begins to work and adds diluted HCl (to decrease the pH) or any other buffering solution to the fluid until it reaches the setpoint, i.e., 7.4, in our case. Depending on the needs of the experiment, the amount of buffering solution can be adjusted.

### 2.9. In Vitro Fluid Dynamic Degradation Test

All samples were degraded in HBSS for 30 days utilizing a fluid dynamic system, as shown in Figure 1, with measurement time points at 1, 2, 7, 14, 21, and 30 days. The degradation of all samples was accelerated by applying a flow velocity of 20 cm/s and a shear stress of 2 dyn/cm^2^. To ensure the samples’ initial cleanliness, they were cleansed in ultrapure water in an ultrasonic bath before the initial fluid dynamic exposure to remove any dust residues. The uncoated AZ31 samples were cleaned in two steps after each exposure to the fluid. They were first cleaned for 5 min in a chromic acid solution to effectively remove the degradation products. They were then cleaned again with ultrapure water in an ultrasonic bath. However, coated samples were cleaned completely in ultrapure water to avoid potential coating degradation caused by the acidic environment of the chromic acid solution.

To ensure precise results, the materials were rigorously dried using a nitrogen flush prior to mass measurements.

## 3. Results and Discussion

### 3.1. Plasma Electrolytic Oxide Formation

**Figure 2:**
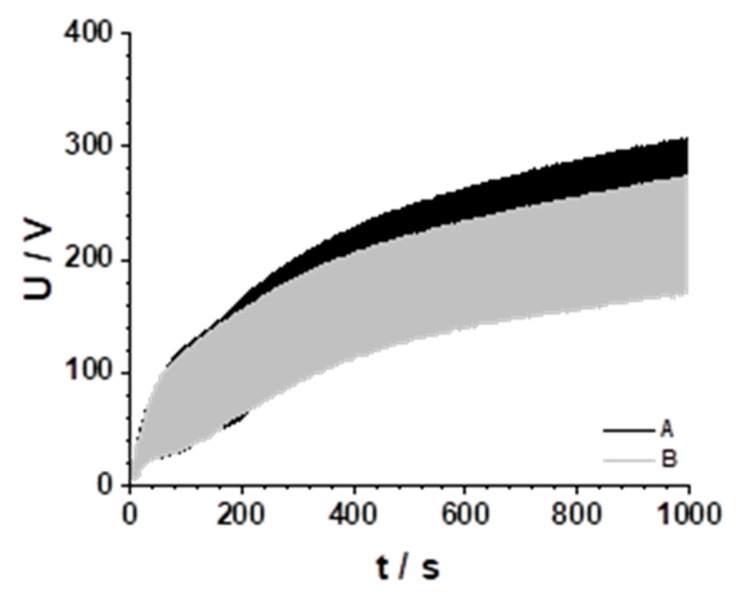
Voltage-time response during PEO for electrolytes A and B.

Figure 2 shows the voltage-time response to the applied current controlled pulse regime of AZ31 in electrolytes A and B. The pulse time on was 0.5 s and the pulse time off was 1 s, therefore Figure 2 shows a compressed view of the pulse regime and not the single pulses. The electrode partially depolarized during the pulse time off.

The necessary overvoltage for plasma electrolytic oxidation is higher in electrolyte A in comparison with electrolyte B. One reason for this could be the different voltage drop across the electrolytes due to their different conductivities. Otherwise, the voltage across the oxide is more than 95%, which means that small differences in oxide thickness must have a higher impact on overvoltages. The PEO process in electrolytes with different KOH contents was described in detail in [28].

### 3.2. Surface Morphology

**Figure 3:**
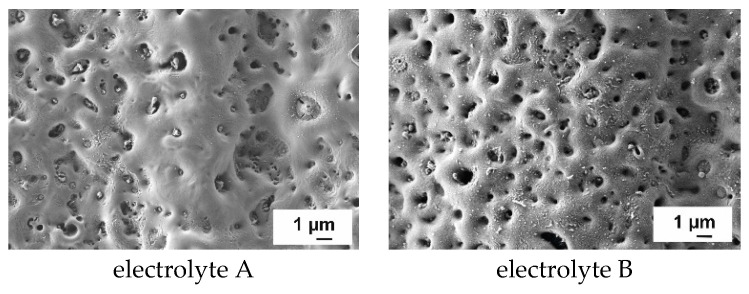
SEM images of PEO layers produced in different electrolytes.

The surface morphologies of PEO layers anodized in electrolyte A and B are shown in Figure 3. The layers show a ceramic-like porous surface structure due to the microdischarges during the PEO process. The pore size varies by a range of few micrometers. On the left side, the PEO layers anodized in electrolyte A show smaller pores, which are partially closed. With increasing KOH concentration, a greater number of pores are opened and the pore size increases. This fact corresponds with the results published in [28].

**Figure 4:**
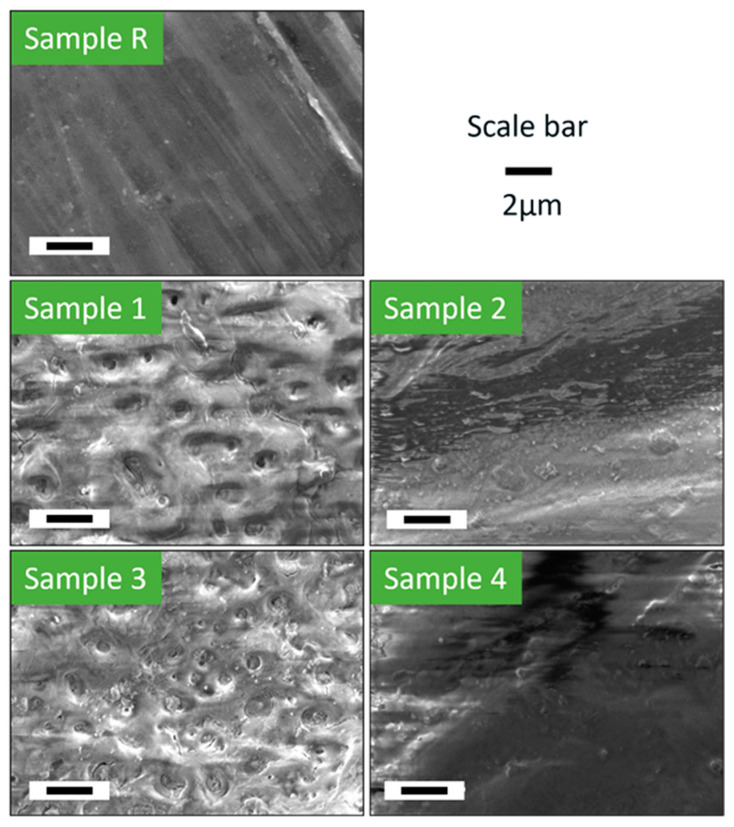
Surface morphology of all samples before degradation test.

Figure 4 shows the surface morphology of all the samples before any degradation testing. The reference specimen (Sample R) without degradation, as expected, displayed only signs of mechanical processing even under high magnification and showed no unusual unevenness. Samples treated with PEO had larger pore size and fewer pores per unit area when KOH (rather than NaOH) was used as the electrolyte in the preparation with NaSiO_3_. Pores can be observed in Samples 1 and 3, but not in Samples 2 and 4 due to the polymer coating over the oxide layer. Sample 3 was prepared similarly to Sample 1, with only the concentration of KOH in the anodization process differing. SEM analysis revealed a greater number of pores in Sample 3 compared to Sample 1, which is due to the lower concentration of KOH used in its preparation. It can be determined that pore size is directly proportional to KOH concentration, while the number of pores is inversely proportional to KOH concentration. Additionally, the porosity of the anodized sample is influenced not just by the type of electrolyte, but also by the quantity of the electrolyte.

**Figure 5:**
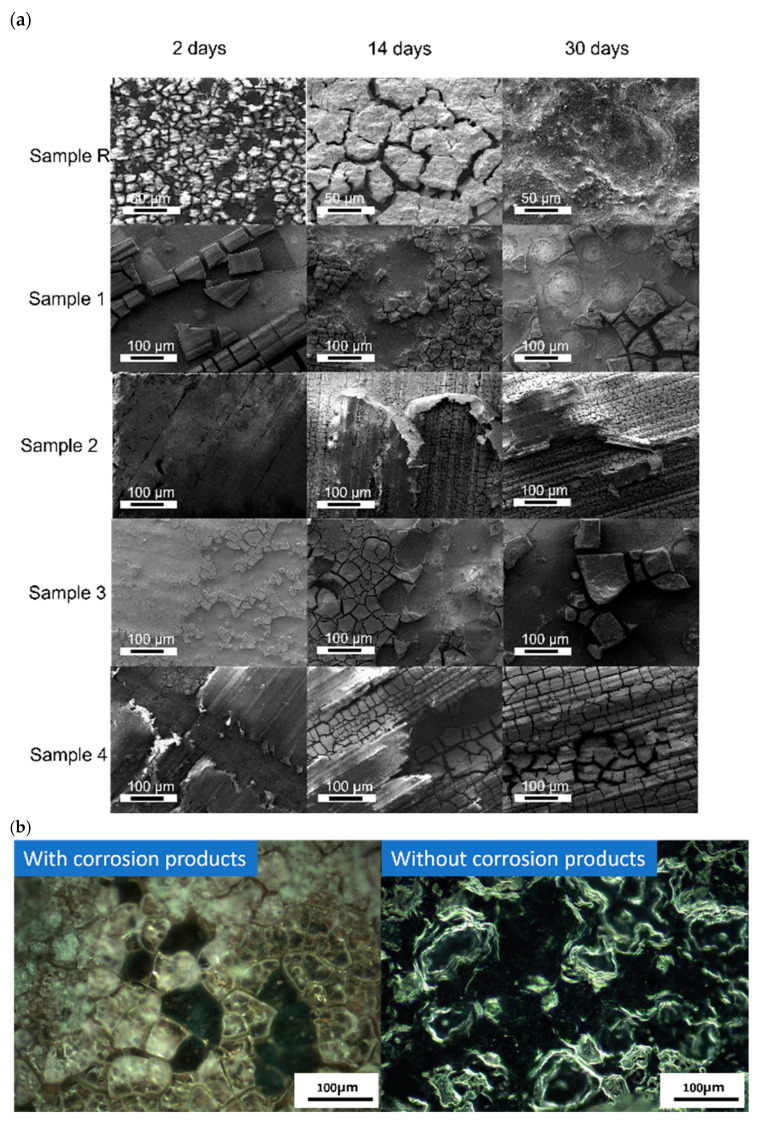
(**a**) Surface morphology of all samples after 2, 14, and 30 days of fluid dynamic degradation test using HBSS. (**b**) Optical microscopic image of Sample R after 14 days of fluid dynamic degradation testing using HBSS at magnification 20×. Only one sample surface is shown here, considering similar crystalline degradation products.

Figure 5a shows the surface morphology of all samples after 2, 14, and 30 days in fluid dynamic circulation.

Sample R began to corrode early on and showed degradation products on the surface. The degradation products were chip-like structures that were smaller after two days of deterioration and changed to larger chips after 14 days. The degradation products covered a significant part of the sample’s surface in both time intervals, but after 30 days of degradation, the regions with and without corrosion products were detected with large and deep pits.

Sample 1 showed that half of the surface was still covered with the cracked PEO layer after two days of fluid dynamic circulation, which was fully removed within the first week. The degradation was similar to that of Sample R after the removal of the oxide layer. Interestingly, white circular traces were observed due to the removal of corrosion products from the surface, which were not present in Sample R.

Sample 2 showed minimal signs of degradation after 2 days due to the polymer coating. The polymer coating was gradually being removed from the beginning, and after 14 days, about half of the polymer coating was removed and a cracked oxide layer was shown. A similar phenomenon was observed, but with less polymer coverage, after the full 30 days of degradation.

Sample 3 showed less resistance to shear stress in a fluidic environment, the coating was removed heavily within the first two days, and the corrosion products were also visible. The coating was removed completely during the first week, and degradation products similar to those appearing on Sample R appeared throughout the degradation period after that, with smaller pits as compared to Sample R.

Sample 4 showed a similar morphology, except with more damage to the polymer coating in the first two days. However, after 14 days of the degradation period, degradation products were also seen where the parts of the cracked PEO layer were removed. After the completion of 30 days, minimal traces of the polymer coating were found on the surface of Sample 4, with the presence of both the PEO layer and corrosion products.

Figure 5b shows the surface of Sample R after 14 days of fluid dynamic degradation in HBSS with and without the degradation products. The crystalline, chip-like degradation products can be seen on the sample’s surface, which were removed after cleaning, leaving pits on the surface.

### 3.3. Electrochemical Behavior

After the PEO, the samples which were produced in two different electrolytes (A and B) were electrochemically tested. The samples were exposed to an electrolyte under open circuit (ocp) conditions over various times. After this exposure, the samples were polarized at −1.7 V in the same electrolyte over 24 h. The polarization was negative from the ocp, resulting in a negative current density, because the cathodic part reaction dominates under the chosen conditions. Since the PEO layer normally has no electronic conductivity, electron transfer reactions like oxygen reduction or hydrogen evolution should not be possible. Only at weak points of the PEO layer, where an electron transfer is possible, can such a reduction take place. Consequently, the consumed negative charge caused by such a reduction is a measurement of the protection level of the PEO layer.

A clear tendency of the consumed charge depending on the exposure time at the OCP is only observed for electrolyte A (Table 3). However, the consumed charge is not strictly proportional to the exposition time. Electrolyte B does not show a clear relation of the consumed charge depending on the exposition time at prior ocp (Table 3). Therefore, the authors assume that the consumed charge depends more on the individual quality of the PEO layer after anodizing, and that this quality dominates the degradation characteristic. Nevertheless, some reduction takes place and also results in a slight increase in the pH after the test.

The same test procedure was carried out on a sample with a PEO layer produced in electrolyte B which was additionally impregnated or coated, respectively, by a polymer (see 2.7. Electrochemical Measurements). In this case, the test solution was HBSS as well as NaOH (see Table 4). In HBSS, the consumed charge is significantly smaller in comparison with the uncoated sample discussed before, which can be explained by the additional protection provided by the polymer film. However, the film was not stable in an alkaline solution, as demonstrated by an increase in the consumed charge.

### 3.4. Degradation Behavior

PEO treated specimens (Sample 1 and 3): Samples 1 and 3 had substantially higher degradation levels at the start of the test, which stabilized to rather steady levels after 7 days (Figure 5). Meanwhile, the corrosion rate of Sample 3 was much higher when compared to the specimens of Sample 1. This is because of the different degradation properties of the anodized layers obtained with electrolytes A and B. When the pH falls below 11.5, the anodized layer becomes porous and chemically unstable, making it susceptible to the corrosive medium. Furthermore, the higher density of magnesium oxide and hydroxide (density MgO: 3.58 g/cm^3^; density Mg(OH)_2_: 2.38 g/cm^3^; density Mg: 1.7 g/cm^3^) than Mg may contribute to anodized layer stress [29], which promotes cracks and corrosion. Moreover, the resulting corrosion of the AZ31 alloy results in local hydrogen evolution, which accelerates the breakdown of the oxide/hydroxide layer [30].

**Figure 6:**
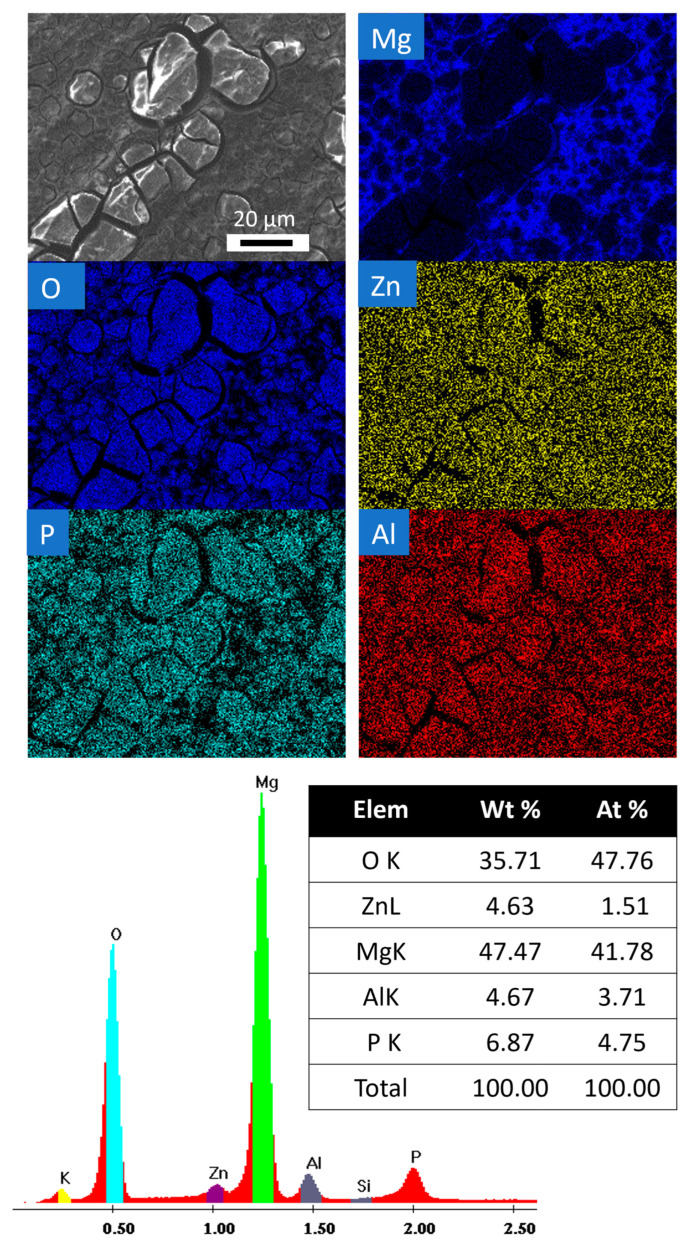
Surface morphology of PEO-treated specimens after the removal of the PEO layer after 2 days of fluid dynamic degradation testing in HBSS. Only one sample surface is presented here considering the similar degradation products in all samples.

Figure 6 shows the surface morphology with the EDX analysis of the coated samples. All of the samples had similar degradation products following the removal of the PEO layer. The elemental mapping and spectra with the quantification of elements after a two-day degradation period are displayed below. It can be seen that the corrosion product layer is dominated by O and P, whereas the degradation products include less magnesium. Additionally, these corrosion products demonstrate that the oxide layer has already been eliminated from these areas during the first two days.

**Figure 7:**
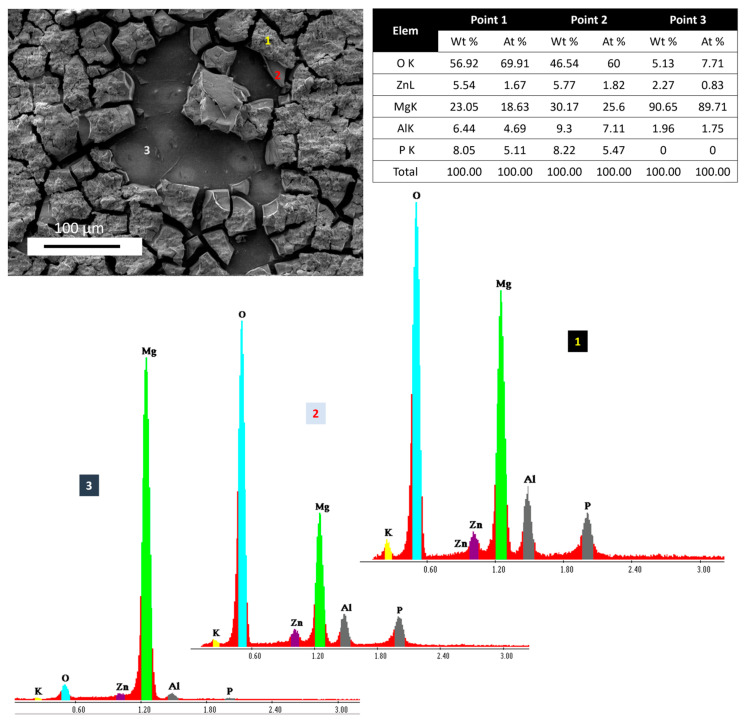
Surface morphology of sample R after 14 days of fluid dynamic degradation testing. The three layers of degraded surface are labeled as Points 1 (topmost layer), 2 (middle layer) and 3 (lowest layer). The elemental quantifications of the elements are shown as At% and Wt%.

Figure 7 shows the surface morphology and point analysis with the EDX spectra and elemental quantification of the Sample R after 14 days, where the degradation products were observed in two different layers. Point 1 shows the topmost layer of the degradation products, where O was higher in the degradation products as compared to at Points 2 and 3. That means the oxygen is becoming less prevalent as we go deeper into the corroded surface. A similar fact was observed for the Zn, Al, and P for Points 1 and 2. After reaching the third layer, i.e., Point 3, the percentage composition of all these elements is reduced significantly. It can be seen that P is not present at the layer of Point 3, demonstrating the absence of any phosphates at this layer. Similar kinds of degradation products were observed in PEO-treated samples as well after the removal of the oxide layer from the surface. On the other hand, the Mg level was lesser at Point 1 as compared to the levels at Points 2 and 3. The Wt% and At% values of Mg demonstrate that, if we go deeper onto the surface, the Mg will become higher.

PEO-treated specimens with polymer coating (Sample 2 and 4): Samples 2 and 4 showed a slower degradation rate as compared to Samples 1 and 3 due to the polymer coating. This served as a barrier between the corrosive medium and the oxide layer of the sample. Initially, only the hydrolysis of the resomer, which is made up of the PLGA, was the reason for the degradation, as the PLGA’s chemical characteristics allow for hydrolytic decomposition by de-esterification [31].

**Figure 8:**
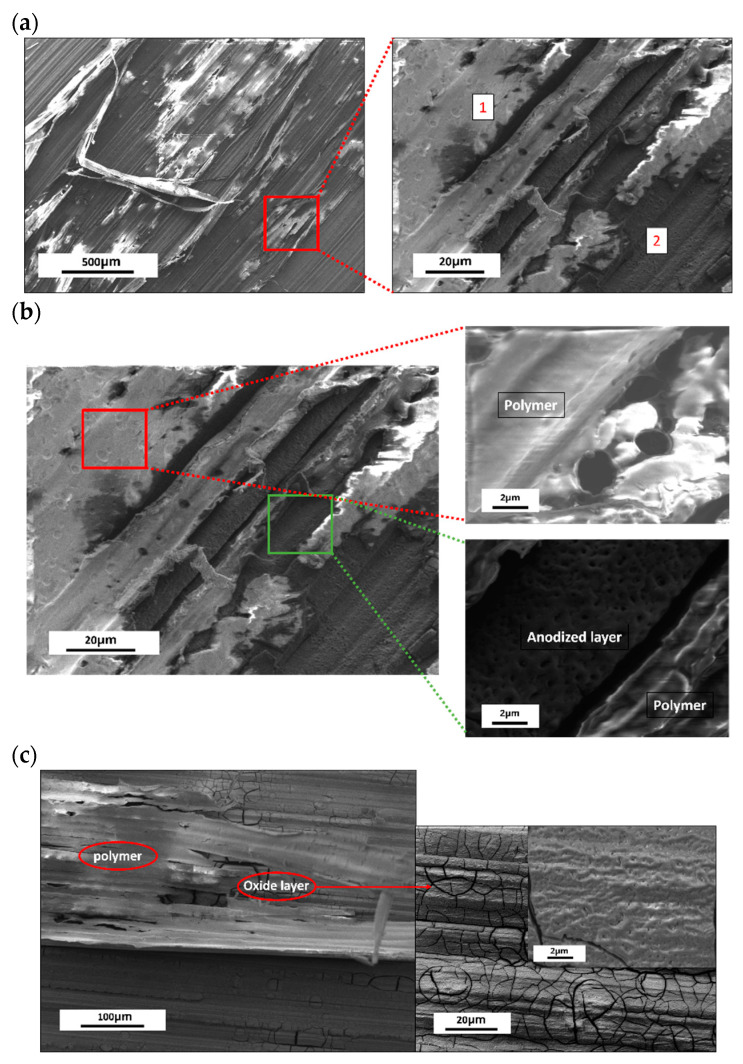
Degradation behavior of polymer-coated samples: (**a**) Degradation behavior after 2 days; (**b**) Magnified images of Points 1 and 2 shown in (**a**); (**c**) Degradation behavior after 7 days.

**Figure 9:**
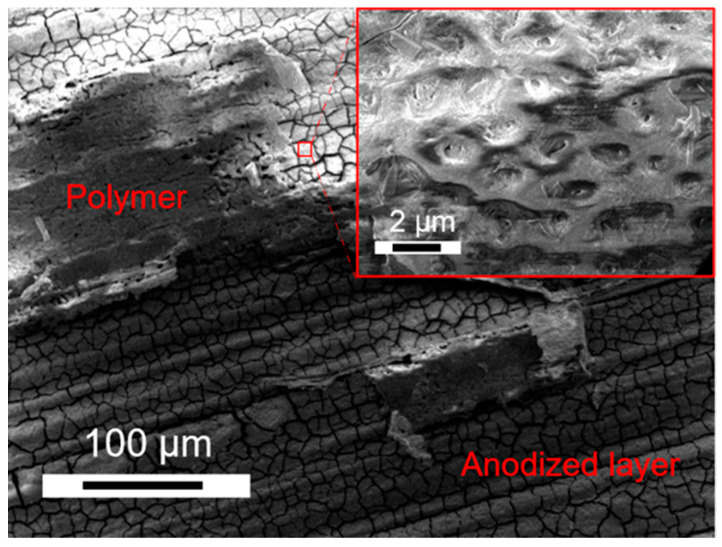
Surface morphology of Sample 2 after 30 days of degradation test.

Figure 8 shows the degradation pattern in the polymer-coated samples, where Point 1 shows the polymer on the surface and Point 2 shows the anodized AZ31 surface after the removal of the polymer coating from this part of the sample. With the passage of degradation time, more and more of the sample’s surface was free from the polymer and the anodized layer was exposed. Then, this anodized layer cracked, as explained above, gradually removing itself from the sample’s surface. After the bulk material is exposed, the degradation products were formed, which were similar to those in Figure 4. In general, Sample 2 had more traces of polymer coating after the 30 days of fluid dynamic degradation, whereas both Samples 2 and 4 have a cracked anodized layer that is still present (Figure 9) and rarely presented with corrosion products due to the removal of this oxide layer.

### 3.5. Surface Wettability

**Figure 10:**
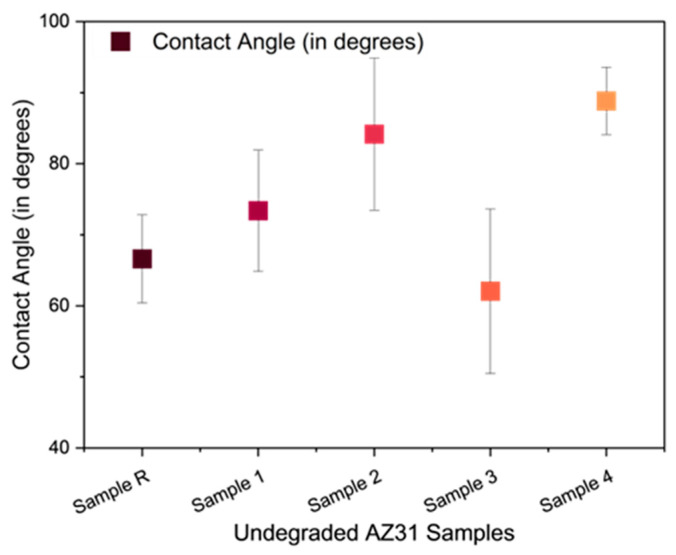
CAs of all samples.

Figure 10 shows the static water contact angles of all samples, including treated and untreated samples. The PEO treatment changed the surface wettability of the AZ31 surface as expected. However, the electrolytes also had an influence on the shift in the wettability towards hydrophobic and hydrophilic regions. Electrolyte A shifted the wettability towards being hydrophilic by increasing the contact angle to 73.4° (±8.5), whereas electrolyte B shifted the wettability towards the hydrophilic region, making Sample 3 more wettable by decreasing the contact angle to 62.0° (±11.5). Samples 2 and 4 (the polymer-coated forms of Samples 1 and 3, respectively) showed the increased contact angles because of the polymer coating, i.e., 84.2° (±10.7) and 88.8° (±4.7), respectively. The resomer coating demonstrated similar behavior on anodized samples. The water contact angle of PLGA in the composition of the used polymer has been reported as 88.5° [32], which is close to the values of the contact angles of Sample 2 (84.2°) and Sample 4 (88.8°).

### 3.6. Mass Loss and Corrosion Rates

**Figure 11:**
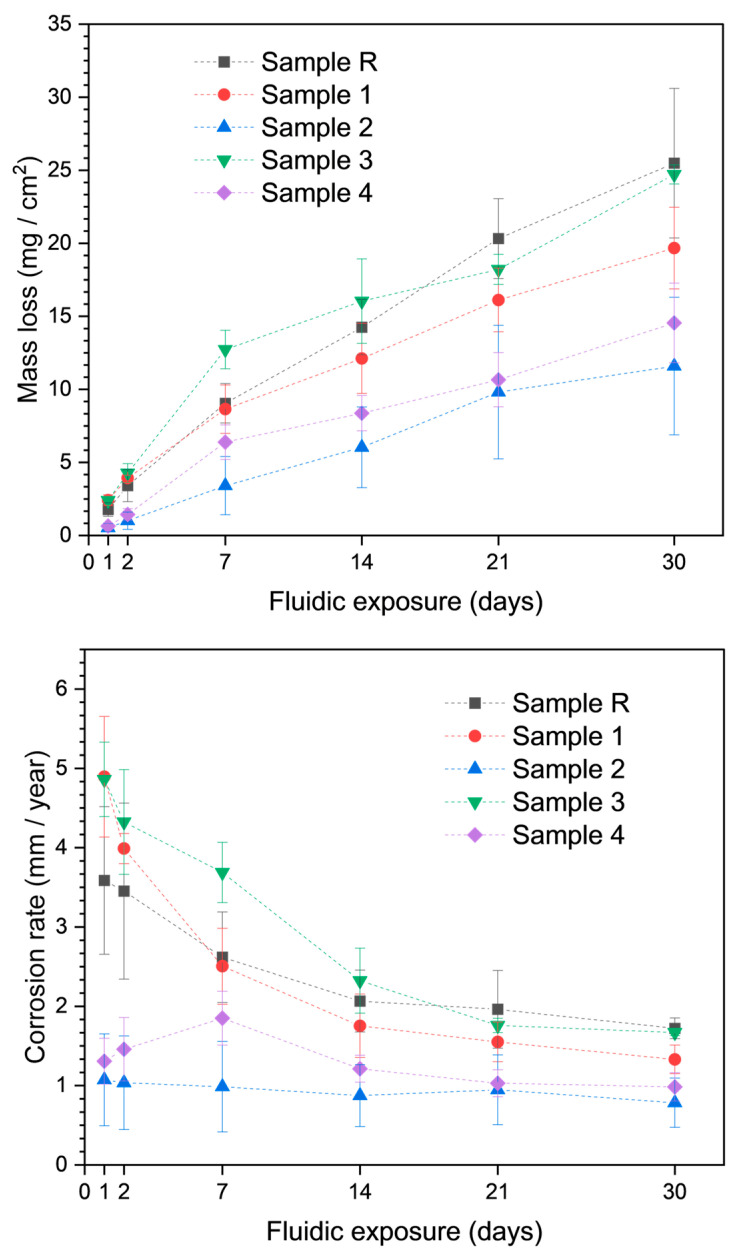
Mass loss and corrosion rates of all samples.

Figure 11 depicts the MLRs and CRs of AZ31 samples during the 30-day fluid dynamic experiment. The results show that both anodizing time and the drying process have an effect on degradation. For example, among non-polymer-coated anodized samples, Sample 3 exhibited higher MLR and CR after 30 days than Sample 1.

Initially, the anodized samples (Samples 1 and 3) showed somewhat higher MLRs than the standard AZ31 surface. This is attributed to the partial removal of the anodized coating over the initial two days. Throughout the experiment, the MLRs of the polymer-coated anodized samples (Samples 2 and 4) were lower than those of any other sample. The anodized layer could not be removed because of the polymer coating. The removal of the oxide layer from the AZ31 surface is the main reason for the elevated ML in anodized samples devoid of a polymer coating.

MLRs and CRs were decreased in both polymer-coated samples, with Sample 2 showing the lowest levels of both among the samples. Polymer-coated samples exhibited consistent behavior and did not exhibit as much fluctuation in MLRs as anodized samples (with the exception of Sample 4’s first week of degradation). The consistent corrosion resistance of Samples 1 and 2 shows that electrolyte A is better suited for obtaining higher corrosion-resistant oxide layers on the AZ31’s surface by PEO treatment. This also conforms to the fact that, for the best results, KOH and Na_2_SiO_3_ should be used in a 1:2 ratio.

## 4. Conclusions

Plasma electrolytic oxidation is a powerful method for increasing the corrosion resistance of magnesium and its alloys. Using a fluid dynamic test, we investigated the accelerated deterioration of PEO-treated AZ31 samples in this study. The results revealed that PEO-treated AZ31 samples were substantially more corrosion-resistant than untreated ones. The best corrosion resistance was demonstrated by samples that were treated using 5 g/L KOH and 10 g/L NaSiO_3_. This result also suggests that the optimum concentrations of the two electrolytes are 1:2. Moreover, the corrosion resistance of the PEO-treated samples was increased even more by applying a polymer (resomer) coating to the anodized surface.

According to the findings of this study, PEO-treated AZ31 with a polymer coating is a promising candidate for biodegradable implant applications. However, more research is needed to assess the long-term performance of this material in vivo. Additionally, it is important to investigate how PEO treatment affects the mechanical characteristics of AZ31 samples.

## Figures and Tables

**Table 1:** Chemical composition in Wt% according to DIN 1729-1 [24].

Al	Zn	Mn	Si	Fe	Cu	Ni	Mg
2.5–3.5	0.5–1.5	0.05–0.4	≤0.1	≤0.03	≤0.1	≤0.0005	Balance

**Table 2:** Composition of the electrolytes.

Electrolyte	KOH (g/L)	Na_2_SiO_3_ (g/L)
A	5	10
B	10	10

**Table 3:** Parameters of the electrochemical tests consist of ocp and potentiostatic measurement in HBSS of the pure PEO layer anodized in electrolyte A and B.

Electrolyte	A	B	A	B	A	B
exposition time at ocp/h	0		1		10	
consumed charge over 24 h at −1.7 V/mAs	−10.7	−17.2	−11.2	−6.8	−19.7	−2.1
pH measured at the start	7.1					
pH measured at the end	8.1	7.9	8.3	8.3	8.2	8.1

**Table 4:** Parameters of the electrochemical test consist of ocp and potentiostatic measurement in HBSS and 0.1 M NaOH of the impregned PEO layer anodized in electrolyte B.

	HBSS	NaOH	HBSS	NaOH	HBSS	NaOH
exposition time at ocp/h	0		1		10	
consumed charge over 24 h at −1.7 V/mAs	−4.6	−35.3	−5.4	−47.4	−3.6	−55.7
pH measured at the start	7.1	12.9	7.1	12.9	7.1	12.9
pH measured at the end	7.4	12.8	7.8	12.8	7.9	12.4

## Data Availability

The raw data supporting the conclusions of this article will be made available by the authors on request.
